# Cochrane's COVID‐19 Living Systematic Reviews: A Mixed‐Methods Study of Their Conduct, Reporting and Currency

**DOI:** 10.1002/cesm.70024

**Published:** 2025-03-28

**Authors:** Kevindu De Silva, Tari Turner, Steve McDonald

**Affiliations:** ^1^ Cochrane Australia, School of Public Health and Preventive Medicine Monash University Melbourne Victoria Australia

**Keywords:** Cochrane, COVID‐19, living systematic reviews

## Abstract

**Background:**

Living systematic reviews (LSRs) should provide up‐to‐date evidence for priority questions where the evidence may be uncertain and fast‐moving. LSRs featured prominently during COVID‐19 and formed part of Cochrane's response to the pandemic. We conducted a mixed‐methods study to describe the characteristics of Cochrane's COVID‐19 living reviews, determine the currency of the included evidence, and evaluate authors' experiences in conducting and publishing these reviews.

**Methods:**

We identified living reviews of COVID‐19 from the *Cochrane Database of Systematic Reviews* and extracted data on the number of versions published and publication timelines. We assessed the currency of evidence by comparing studies included in the reviews against a comprehensive list of studies maintained for the Australian living guidelines for COVID‐19. The qualitative component involved semi‐structured interviews with review authors to identify the barriers and enablers to conducting, reporting and publishing living reviews.

**Findings:**

Cochrane published 25 COVID‐19 living systematic reviews. Half of these reviews had not been updated when assessed in June 2023 and only four had been updated more than once. A total of 118 studies were included in the living reviews. We estimated that an additional 119 studies were available and potentially relevant for inclusion. Interviews with six authors indicated that publication timelines were reduced by editorial delays, loss of funding, waning commitment, and the burden of screening search results. An inability to communicate the living status of reviews in the Cochrane Library was a common frustration for many authors. Although authors felt the conclusions of their reviews were still current, only one living review communicated its updated status and made new evidence accessible after the review was published.

**Conclusions:**

Maintaining and communicating the currency of Cochrane's COVID‐19 living systematic reviews was not feasible for many author teams because of author‐side, editorial and platform barriers.

## 1 Introduction

Systematic reviews are abundant in health research but can soon become out of date as new research is published [1, 2]. Living systematic reviews (LSRs) were conceived to address this issue, particularly for priority topics where the evidence is uncertain and there is a known pipeline of new studies [3, 4]. Cochrane guidance states that LSRs require continual, active monitoring of research and should immediately include important new evidence [5]. These reviews should also be accompanied by up‐to‐date information about the review's status, including whether new evidence has been identified, to ensure users benefit from the living approach.

Cochrane guidance recommends that Cochrane LSRs state the review is living in the abstract, plain language summary and objectives, though not explicitly in the title. It also suggests authors either update their reviews on a fixed‐interval schedule or when new evidence is likely to impact review conclusions. Although a new chapter of the *Cochrane Handbook for Systematic Reviews of Interventions* is planned, we are not aware of guidance from groups other than Cochrane that covers the conduct of living reviews. The extension of the PRISMA 2020 statement for living systematic reviews (PRISMA‐LSR) was published in November 2024, which provides guidance on reporting of LSRs [6].

The prospect of keeping up with rapidly evolving evidence made living reviews a popular synthesis type during the COVID‐19 pandemic [7]. Despite the conceptual advantages of living over standard systematic reviews, studies have shown that authors struggled to maintain their currency, limiting their usefulness [8, 9, 10]. As a leading producer of systematic reviews and pioneer of living review methods, Cochrane embraced the idea of publishing LSRs during the pandemic.

The study addressed three research questions: (1) what are the characteristics of Cochrane's COVID‐19 living reviews, (2) what is the currency of the included evidence, and (3) what were the authors' experiences in conducting, reporting and publishing these reviews.

## 2 Methods

### 2.1 Study Design

This was a mixed‐methods design comprising a cross‐sectional study of the characteristics and currency of Cochrane COVID‐19 living reviews, and a qualitative exploration of authors' experience conducting them. Where applicable, reporting of the methods and results comply with the consolidated criteria for reporting qualitative studies (COREQ) checklist (see Appendix A) [11]. Ethics approval was provided by Monash University Human Research Ethics Committee (Project ID: 37953).

### 2.2 Selection Criteria

Reviews were eligible if they were described as living or followed a living approach. We searched the *Cochrane Database of Systematic Reviews* on 1 June 2023 for reviews of COVID‐19 or SARS‐CoV‐2 that included the word ‘living’ anywhere in the text: (covid* or sars* or coronavirus):ti and living (all text). Retrieved reviews were independently screened by two researchers (KDS, SM) with any disagreement resolved in consultation with a third (TT).

### 2.3 Quantitative Data Collection and Analysis

We extracted data on the following review characteristics: number of updates, communication of living status (e.g., access to evidence identified post submission), and submission/publication timelines (see Appendix B). Data were collected in Excel by a single researcher (KDS) after piloting on three reviews. Data were directly extracted from the reviews except for information on submission dates which was provided by Cochrane's Central Editorial Service.

Evaluation of currency was restricted to intervention and prevention reviews since we had access to a comprehensive list of randomised trials maintained for the Australian living COVID‐19 guidelines [12]. (Vaccines, diagnostic tests and risk factors were outside the scope of these guidelines.) The list of trials was categorised by treatment (e.g., type of drug) and included information on source, publication date and sample size.

For each living review, we checked the included and excluded studies against our list of COVID‐19 trials and noted those studies that were potentially available (and which could have been expected to be found by the review's search) but had yet to be included in the review or listed as waiting assessment. Trials published as preprints were deemed to be available from the publication date of the preprint. Reviews that were retired from living mode before June 2023 were excluded from this phase of the analysis. To avoid overestimating the number of studies available for inclusion in each review, we excluded the following: trials with active comparators (e.g., ivermectin vs doxycycline) or combined interventions (e.g., doxycycline and zinc); trials reported only as media releases; and trials that had been retracted or concerns expressed about the validity of the data.

Data were presented as simple descriptive statistics (e.g., frequencies, medians).

### 2.4 Qualitative Data Collection and Analysis

Participants were eligible if they were an author on at least one COVID‐19 living review. We invited all 15 contact authors (several of whom were the contact author of more than one review) to participate in interviews and to share the invitation with their co‐authors, as appropriate. We sent reminder emails to authors who had not replied after 3 weeks. A participant information sheet explained that agreeing to be interviewed was considered informed consent.

We conducted semi‐structured interviews over Zoom between June and August 2023 using an interview schedule that was developed afresh by the study investigators and included questions tailored to the author's role in the review and their responses to earlier questions (see Appendix C). Questions focused on enablers and barriers to conducting and publishing living reviews, and what changes could improve their experience. Interviews were conducted by KDS with at least one other present (SM or TT) and lasted around 45 min. Interviews were video‐recorded and transcribed using Temi, an online transcription service. KDS reviewed and corrected the transcripts – respondent validation was not conducted.

Interview data were thematically analysed using an inductive approach in NVivo. Two transcripts were independently coded by KDS and TT to generate initial codes, which were then discussed and collapsed into themes. KDS coded the remaining transcripts and discussed any issues with TT.

### 2.5 Reflexivity Statement

KDS is a medical student with no previous experience with living reviews. TT was involved in developing the initial concept of living systematic reviews. TT and SM helped develop Cochrane's LSR guidance and are involved in Cochrane living reviews in areas unrelated to COVID‐19. Through our involvement in the Australian living COVID‐19 guidelines, we were aware of the living reviews of COVID‐19 published by Cochrane and how infrequently some were being updated. Part of the rationale for this study was about exploring and understanding the relationship between author‐side barriers and organisational/editorial challenges in publishing Cochrane living reviews, with a view to improving their timeliness, usefulness and impact. All authors are supportive of the concept and believe in the value of living systematic reviews, and this may have influenced our research approach and interpretation of the findings.

## 3 Results

### 3.1 Living Reviews

Our search of the *Cochrane Database of Systematic Reviews* identified 40 reviews. Fifteen were excluded because the word ‘living’ was used to describe the population of interest (e.g., people living with dementia) rather than the approach of the review. The 25 eligible COVID‐19 reviews covered topics relating to treatment (16 reviews), prevention (4), diagnosis (5) and aetiology (1) (Table 1) [13, 14, 15, 16, 17, 18, 19, 20, 21, 22, 23, 24, 25, 26, 27, 28, 29, 30, 31, 32, 33, 34, 35, 36, 37]. One review of ivermectin covered both prevention and treatment.

**Table 1:** Topics covered by Cochrane's COVID‐19 living systematic reviews.

Treatment	Prevention
1. Nirmatrelvir‐ritonavir 2. Convalescent plasma 3. Hyperimmune immunoglobulin 4. Systemic corticosteroids 5. Inhaled corticosteroids 6. Treatment of persistent olfactory dysfunction 7. Janus kinase inhibitors 8. Colchicine 9. Neutralising monoclonal antibodies to treat COVID‐19 10. Remdesivir 11. Vitamin D supplementation 12. Interleukin‐6 blocking agents 13. Fluvoxamine 14. Ivermectin 15. Antibiotics 16. Interleukin‐1 blocking agents	1. Ivermectin 2. Efficacy and safety of COVID‐19 vaccines 3. Prevention of persistent olfactory dysfunction 4. Neutralising monoclonal antibodies to prevent COVID‐19
	**Diagnosis**
	1. Thoracic imaging tests 2. Signs and symptoms 3. Routine laboratory testing 4. Rapid, point‐of‐care antigen and molecular‐based tests 5. Antibody tests
	**Aetiology**
	1. Thromboembolism risk in COVID‐19 patients using hormonal contraception

### 3.2 Characteristics of Living Reviews

Half (13/25, 52%) the living reviews were never updated, a third (8/25, 32%) were updated once and only four (16%) updated more than once [14, 32, 33, 35]. In total, there were 44 published versions of these 25 reviews. Two‐thirds (16/25, 64%) of reviews stated in the title or abstract that they were living. As of June 2023, 80% (20/25) of reviews were still identifiable in the Cochrane Library as living (Table 2). Three‐quarters (19/25, 73%) of reviews included a description of the trigger for updating (either fixed‐interval updates or when conclusions changed), but for the remainder this was either unclear or no criteria were provided. A quarter of reviews (6/25, 27%) gave criteria for transitioning out of living mode. See Appendix D for detailed characteristics of included reviews.

**Table 2:** Status of Cochrane's COVID‐19 living systematic reviews.

Characteristic	*n* (%)
**Where is the living approach first mentioned?**	***N*** = **25**
– Title/abstract – Plain language summary, background or objectives	16 (64%) 9 (36%)
**How many times has each living review been updated?**	
– Never updated – Updated once – Updated twice – Updated 3 times – Updated 4 times	13 (52%) 8 (32%) 2 (8%) 1 (4%) 1 (4%)
**Year of first publication**	
– 2020 – 2021 – 2022 – 2023	6 (24%) 10 (40%) 7 (28%) 2 (8%)
**As of June 2023, was the review still in living mode?**	
– Yes – No	20 (80%) 5 (20%)
**For reviews still in living mode, was new evidence identified between submission and publication communicated to readers?**	***N*** = **20**
– Yes – No	16 (80%) 4 (20%)
**For reviews still in living mode, was new evidence identified post‐publication communicated to readers?**	
– Yes – No	1 (5%) 19 (95%)
**For updated living reviews (latest version), did authors describe if changes affected the conclusions?**	***N*** = **12**
– Yes, conclusions changed – Yes, conclusions remained unchanged – No description of how the conclusions changed or if they stayed the same	6 (50%) 2 (17%) 4 (33%)

Although most reviews still in living mode (16/20, 80%) made studies identified between submission to Cochrane and publication in the Cochrane Library available to readers (through the ‘Studies awaiting assessment’ section), only one review put in place a mechanism for readers to access studies identified after publication of the review [13].

Table 3 presents the time elapsed between the search, submission and publication of each version of the review. Around half were submitted within 3 months of the search date (17/32; 53%). For updated reviews, half were published within 12 months of the previous version's publication (9/19; 47%). As of June 2023, a third (7/20; 35%) of reviews still in living mode had not been updated in over a year.

**Table 3:** Publication characteristics of Cochrane's COVID‐19 living systematic reviews.

Characteristic	*n* (%)
**Time elapsed from date of search to submission** – < 3 months – 3–6 months – 7–12 months – > 12 months	***N*** = **32** a 17 (53%) 12 (38%) 2 (6%) 1 (3%)
**Time elapsed from date of submission to publication**	
– < 3 months – 3–6 months – 7–12 months – > 12 months	18 (56%) 4 (13%) 10 (31%) 0 (0%)
**For updated reviews, time elapsed from publication of previous version** – < 6 months – 7–12 months – 13–18 months – > 18 months	***N*** = **19** 5 (26%) 4 (21%) 7 (37%) 3 (16%)
**For reviews in living mode (June 2023), time elapsed from publication of previous version** – < 6 months – 7–12 months – 13–18 months – > 18 months	***N*** = **20** 4 (20%) 9 (45%) 2 (10%) 5 (25%)

^a^ Submission data were not available for 12 of the 44 review versions and so were not included in these analyses.

Across all versions of the reviews, it took around 5 months from the date of search to publication in the Cochrane Library (*n* = 44; median 152 days; IQR = 73–302). Searches were typically re‐run 7 months after the previous search (*n* = 44; median 199 days; IQR = 106–319). Reviews with updates were usually published more than a year after the previous version (*n* = 12; median 410 days; IQR = 168–472). Six reviews indicated a fixed‐interval update schedule of 4 to 6 months; however, only one followed the predetermined schedule [37] – the remainder had either not been updated or a year had elapsed since the most recent version.

Almost all living reviews (18/19, 95%) submitted before October 2021 were published within 3 months of their submission compared with none (0/13) submitted after this date. Three of the 13 review updates submitted after October 2021 were published within 3–6 months and 10 within 6–12 months.

### 3.3 Review Currency

Sixteen reviews of treatment or prevention that were still in living mode in June 2023 were assessed for currency against the list of COVID‐19 randomised trials maintained for the Australian COVID‐19 living guidelines. Of the 241 randomised trials deemed applicable to the inclusion criteria for these reviews, 118 were already included (or listed as excluded) and four were listed as awaiting assessment, leaving 119 that were not included (or listed in the review as excluded). All but one review had studies that were potentially eligible for inclusion. Across the 16 reviews, the median number of studies already included was 5 (IQR = 3–7) and the median number potentially eligible for inclusion was 6 (IQR = 3–13) (Appendix E).

Most (69/119, 58%) of the potentially eligible studies were published after publication of the latest version of the review. We estimate these studies collectively included over 22,000 participants. The remaining studies (49/119, 41%), with one exception, were published between the review's submission and publication date, and represent over 14,000 participants. For one review, we identified a preprint of a trial of 64 participants that was not included (or listed) in the review and which was published before the review's search date. Collectively, these 119 studies thus represent the evidence that could have been included in living reviews had they been updated or updated more frequently.

### 3.4 Experiences of Cochrane LSR Authors

#### 3.4.1 Participants

Fifteen contact authors were invited to take part. One contact author was responsible for five reviews, two were responsible for three reviews each, and one was responsible for two reviews. Seven did not respond and two declined but recommended a coauthor. We interviewed six authors (five of whom were contact authors) who were collectively involved in 14 of the 25 living reviews, covering diagnostic, prevention and treatment topics.

Interviewees' roles included screening, analysing data and review writing. Experience with reviews varied among the interviewees – for example, it was the first time one author had been involved in any type of systematic review, while others were senior authors with substantial review expertise. All interviewees were female and based in Europe. Interviews focused on motivations for living reviews, enablers and barriers, and what changes could improve the author experience. The main themes to emerge are summarised in the sections below.

#### 3.4.2 Authors Believed Living Reviews Would Have a Greater Impact

Authors created their reviews in living mode in the belief it was the best way to maintain up‐to‐date evidence syntheses. By providing information quickly, clinicians could rapidly transfer recommendations into clinical practice, leading to improved care, avoiding harm and reducing research waste.

“We wanted a high‐quality approach of finding out what is the actual evidence that we've got and keep updating it regularly when there's new, significant information available.”

– Participant 1

However, authors were not confident that their reviews were being widely used. Participant 3 noted that reviews were used most in their own country because of their affiliation with their national COVID‐19 guidelines. Participant 4's experience was different – their government was largely unaware of their living review. Participants also felt that living reviews should be considered more important than standard reviews but were not.

“Not all national policymakers were aware of what we were doing… The reviews, once they were out, were used as just another study and not as the most up‐to‐date evidence at that point in time.”

– Participant 4

Despite experiencing several barriers when conducting and publishing their reviews, authors still retained confidence in the value of living reviews.

“In a pandemic situation, and in a rapidly changing situation, it still has its value. You need it. You have to very frequently update the systematic review. A living systematic review is probably the most efficient way to do that.”

– Participant 4

#### 3.4.3 Maintaining and Communicating Review Currency was Challenging

Authors were frustrated that they could not easily communicate the currency of their living review. Most authors acknowledged that searches were out of date but believed the conclusions were still current. Some authors noted that the interventions being reviewed had become clinically irrelevant and therefore if the conclusions were out of date, it was not a point of concern. However, they struggled to communicate these status updates to readers.

“We do not need further updates, but there is no way to show that, and nobody knows what we are thinking.”

– Participant 2

Authors also believed that living reviews were ultimately not that different from standard reviews.

“We came to the conclusion that so far, pretty much every living systematic review is just a review that's updated here and there.”

– Participant 6

#### 3.4.4 Strong Relationships Increased the Feasibility of the Living Approach

Expertise among the author team increased efficiency. Many teams involved senior researchers with experience in both standard and living Cochrane reviews. When questions arose, junior researchers consulted these senior researchers instead of methods guidance documents. Conducting searches was a straightforward part of the process when teams included information specialists.

“The group that we were working with was very experienced in doing diagnostic reviews. So that was definitely something that made things very easy.”

– Participant 5

“I think with the first review, it was kind of slowed by us being a new team, most of us inexperienced with that kind of approach.”

– Participant 3

Participants 2 and 6 highlighted that a longstanding relationship and direct communication with Cochrane's central editorial team ensured a smooth and rapid progress through the editorial process. However, this experience was not universal and other authors' teams frequently encountered editorial delays.

“Good communication between the author team and editorial team is really important.”

– Participant 2

#### 3.4.5 Rapid Publication Cycles had Limited Sustainability

The pressing nature of the pandemic plus the anticipated benefits of living reviews meant that review authors were willing to invest time in producing up‐to‐date evidence syntheses. However, this sense of urgency diminished as the pandemic unfolded.

“As the pandemic evolved, normal work resumed, and people were going back to what they were doing before everything started. It became a burden on people's workload to be able to keep going at the speed that we were going.”

– Participant 5

Sustaining the commitment was especially challenging for clinician‐authors who had to balance review work with clinical duties. Several authors recognised the importance of having team members who were continually involved in monitoring the evidence base. Otherwise, the efficiency gains of living reviews over standard reviews were lost.

Withdrawal of funding meant author teams could not maintain the same level of commitment as the pandemic evolved. In several cases, authors reverted to using intramural funding to maintain reviews in living mode, at which point the reviews were treated as just another research task. Some authors retired their reviews because of funding issues.

“Funding would have made a difference for the prioritisation of these reviews.”

– Participant 4

Authors believed that automation was key to reducing workload in maintaining living reviews.

“Everything else that comes before [data analysis] is quite technical and repetitive and could be hopefully outsourced to machines so that we can save our time for where we can really make a difference.”

– Participant 5

The volume of low‐quality studies was an issue for many authors, especially those involved with diagnostic test accuracy reviews. Although the number of high‐quality studies increased during the pandemic, the number of low‐quality studies increased more rapidly.

“We gave up and said ‘Well, this is not working: people are just doing the same stuff all over again and we're not improving what we can tell people’. So we retired our review.”

– Participant 5

Authors noted the importance of peer reviewers and editors acquiring familiarity with the review topics.

“It would be great to have the same editorial team for every update, so people are in the material, so they don't need much time to understand the whole process and background of the review.”

– Participant 2

Several authors noted that editorial processes were more efficient early in the pandemic, before being subject to increasing delays. One author recalled peer review taking half a year, reducing the currency of their reviews.

“We've done all of the work in a far shorter time than the editorial process, so it was up to date, but by the time it gets published, it's out of date again and we're already doing the update again.”

– Participant 1

“I think for Cochrane, it's very fast, but it's too long. Still too long.”

– Participant 2

## 4 Discussion

### 4.1 Main Findings

We investigated the characteristics and currency of COVID‐19 living reviews published by Cochrane and interviewed authors to explore their experiences. Although Cochrane's guidance for living reviews emphasises the timely incorporation of new evidence, 13 of the 25 COVID‐19 living reviews were never updated, eight were updated once and only four were updated more than once. This is despite the availability of new studies for nearly all reviews. Communicating the status of living reviews to readers was considered problematic by author teams as there is currently no simple mechanism to convey this information via the Cochrane Library platform.

At the outset authors expected they would maintain their reviews by regularly incorporating new studies. Our finding that only half the living reviews were ever updated might appear low given the circumstances of the pandemic but is double the number reported by Chen et al in their analysis of 64 COVID‐19 living reviews [8], and higher than the 38% reported by Zheng et al in their bibliometric analysis of 213 living systematic reviews across all topic areas [10]. This suggests that, despite good intentions, Cochrane author teams struggled to maintain reviews in living mode. Authors alluded to several barriers including waning commitment as the pandemic unfolded; minimal changes to the underlying evidence despite the accumulation of new studies; loss of funding; and the burden of screening, especially for diagnostic test reviews. These barriers help explain why many living reviews initiated at the start of the pandemic were not updated and are not necessarily generalisable to other living reviews in the Cochrane Library.

Living reviews that were published more than once took on average a year to be updated, though updates earlier in the pandemic were published faster. Structural and organisational changes to Cochrane's editorial processes were cited as a significant contributor to delays in the publication of updates after October 2021.

Many living reviews indicated that updates would be published when new evidence resulted in changes to the conclusions of the review. Thus despite reviews being infrequently updated, and many being a year or more out of date, authors considered the conclusions were still current. However, readers have no way of being reassured that this is the case—they must rely on the authors' pledge that an update will be published promptly when new evidence affects the conclusions. The exception to this was the review of nirmatrelvir combined with ritonavir (Paxlovid) [13] which used a spreadsheet on Open Science Framework to transparently report the living evidence surveillance process between published versions [38].

Our estimate that by June 2023 there were 119 studies awaiting inclusion in reviews highlights a particular problem with Cochrane's COVID‐19 LSRs—reviews may not be out of date in terms of their conclusions but nonetheless remain ‘living’ despite rarely being updated and despite the availability of many new studies. Readers might reasonably expect living reviews to incorporate all relevant studies in a timely way.

### 4.2 Implications for Cochrane Living Reviews

Timely communication around living reviews and changes in their status is essential if these reviews are to fulfil their purpose of keeping readers informed in areas where evidence is evolving rapidly. Having reviews in the Cochrane Library that continue to be described as living, despite never or infrequently being updated, potentially undermines credibility and trust readers have in Cochrane reviews. This risk to credibility potentially extends to all living reviews if readers are unable to discern if a review is actively being maintained in living mode and thus whether the conclusions of the review are up to date.

Fixed‐interval update schedules could minimise this risk, but only if review authors keep to the schedule and journals have mechanisms that support the publication of regular updates. The model adopted by *Annals of Internal Medicine* during the pandemic of publishing regular updates, either in the form of short alerts (conclusions unchanged) or major updates (conclusions changed), provides a blueprint for how the status of living reviews can be effectively communicated to readers [39, 40].

To the frustration of authors we interviewed, there is currently no simple mechanism in the Cochrane Library that enables authors to easily communicate updates in the status of the review or to signal that a review has been retired from living mode. The challenges of managing updates and keeping readers informed of the review status were a feature of the experiences of maintaining the Cochrane COVID‐19 living reviews of convalescent plasma and corticosteroids [41].

Having clear and consistent criteria for labelling or describing a review as ‘living’ and being diligent in removing this label or description once a review transitions out of living mode would avoid the presence of “zombie” living reviews in the Cochrane Library—reviews that are living in name only. Managing the status of these reviews requires a more dynamic online publishing platform, transparency from authors about when their review ceases to be living, and regular monitoring by Cochrane's central editorial team to judge when a living status is no longer appropriate.

Cochrane has previously recognised the challenge of communicating the update status of reviews (whether standard or living) and proposed the Updating Classification System as a solution in 2016, but it was never implemented [42]. The updating classification system would have allowed authors to indicate their review's update status as ‘No update planned’, ‘Up to date’ or ‘Update pending’ with a rationale for their selected status. A similar system could help LSR authors communicate the status of their review at each update.

In the absence of a reporting system for living reviews in the Cochrane Library, the living Cochrane review of electronic cigarettes for smoking cessation shows how the status of living reviews can be communicated to readers via the version history section of the review. Details of new studies identified from monthly searches are listed as amended events in the version history before being incorporated in a future update [43, 44]. Wagner et al also recommend exploring mechanisms outside of the Cochrane Library for communicating regular updates, such as review‐specific websites or the publication of non‐peer‐reviewed preprint versions [41].

Inconsistent reporting of changes in conclusions suggests Cochrane's guidance for living reviews should be updated to recommend authors explicitly report changes from one version to the next, something advocated by Iannizzi et al. [45] In addition to the description that appears in the version history section of the review, Cochrane could consider recommending that living reviews include a statement in the abstract summarising the main changes from the previous version.

The recent centralisation of Cochrane's editorial processes provides an opportunity to address several of the shortcomings with living reviews identified by this study and to implement consistent reporting standards. The impetus for this will only increase following the recent publication of the extension of the PRISMA 2020 statement for living systematic reviews (PRISMA‐LSR) [6].

### 4.3 Strengths and Limitations

This is the first comprehensive assessment of Cochrane's COVID‐19 living reviews. The inclusion of a qualitative component allowed us to explore the barriers and facilitators of producing living reviews during the pandemic and identify areas where Cochrane can improve its processes. The only previous qualitative study was a pre‐pandemic evaluation of several pilot Cochrane living reviews [46]. This evaluation also found workload, editorial challenges and publication delays to be among the most pressing challenges for authors.

Because this study focused on Cochrane living reviews of COVID‐19, the findings may have limited generalisability to LSRs published outside of Cochrane or to topics unrelated to the pandemic. In contrast to other living reviews published by Cochrane, the emphasis on speed, the unpredictable nature of the pandemic, and the waning motivation as the threat of the pandemic subsided has likely resulted in more reviews being initiated (and left) in living mode than would otherwise be expected.

The relatively small number of review authors interviewed was a limitation, although over half the reviews in the sample were represented. It was unclear if data saturation was achieved; however, the convergence of themes in the interviews and consistency of these themes with the experiences of LSR teams described in previous research with Cochrane authors increases our confidence in the results [45, 46].

## 5 Conclusion

Maintaining the currency and communicating the update status of Cochrane's COVID‐19 living systematic reviews was not feasible for many author teams. In many cases, the urgency to conduct updates subsided and author teams considered reviews current despite the publication of new studies. Although the circumstances of the pandemic limit the generalisability of findings to other living reviews, this study highlights the need for Cochrane to carry out internal housekeeping of its collection of COVID‐19 reviews to ensure only those reviews that remain in living mode are described as such in the Cochrane Library. Beyond the influence of the pandemic, this study highlights several challenges faced by author teams, including internal organisational and editorial barriers and limitations with the existing publishing platform that apply equally to Cochrane living reviews unrelated to COVID‐19.

## Author Contributions

**Kevindu De Silva:** conceptualization, data curation, formal analysis, investigation, methodology, writing – original draft, writing – review and editing. **Tari Turner:** conceptualization, formal analysis, investigation, methodology, supervision, validation, writing – review and editing. **Steve McDonald:** conceptualization, formal analysis, investigation, methodology, supervision, validation, writing – review and editing.

## Ethics Statement

Ethics approval was provided by Monash University Human Research Ethics Committee (Project ID: 37953).

## Conflicts of Interest

TT and SM developed Cochrane's guidance for living systematic reviews, evaluated Cochrane's pre‐pandemic LSR pilot, and are co‐authors of Cochrane living reviews unrelated to COVID‐19. Both have Cochrane editorial roles – TT is a member of the Cochrane Library Editorial Board and SM is a member of the Cochrane search peer review panel. All authors are associated with the Australian Living Evidence Collaboration.

## Peer Review

The peer review history for this article is available at https://www.webofscience.com/api/gateway/wos/peer-review/10.1002/cesm.70024.

## Supporting information

Supporting information.

## Data Availability

Data are available from the corresponding author upon request.
