# Supplementary material for: Cochrane's COVID‐19 Living Systematic Reviews: A Mixed‐Methods Study of Their Conduct, Reporting and Currency
Source: Cochrane Evid Synth Methods. 2025 Mar 28;3(3):e70024. doi: 10.1002/cesm.70024 (PMC12245076; doi:10.1002/cesm.70024)
Supplement: Supplementary file 1 — Supporting information. [file CESM-3-e70024-s001.pdf]

Supplementary material

De Sliva, K. et al. Cochrane's COVID-19 living systematic reviews: a mixed-methods study of their conduct, reporting and currency.

List of appendices

1. Appendix A: COREQ checklist
2. Appendix B: summary of characteristics extracted
3. Appendix C: interview outline and questions
4. Appendix D: characteristics of included living reviews
5. Appendix E: characteristics of randomised trials eligible for inclusion

APPENDIX A

Consolidated criteria for reporting qualitative studies (COREQ): 32-item checklist

De Sliva, K. et al. Cochrane's COVID-19 living systematic reviews: a mixed-methods study of their conduct, reporting and currency

No. Item	Guide questions/description	Section #
**Domain 1: Research team and reflexivity**		
Personal Characteristics		
1. Inter viewer/facilitator	Which author/s conducted the interview or focus group?	2.4
2. Credentials	What were the researcher's credentials? E.g. PhD, MD	2.5
3. Occupation	What was their occupation at the time of the study?	2.5
4. Gender	Was the researcher male or female?	N/A
5. Experience and training	What experience or training did the researcher have?	2.5
Relationship with participants		
6. Relationship established	Was a relationship established prior to study commencement?	N/A
7. Participant knowledge of the interviewer	What did the participants know about the researcher? e.g. personal goals, reasons for doing the research	[Participant information sheet]
8. Interviewer characteristics	What characteristics were reported about the inter viewer/facilitator? e.g. Bias, assumptions, reasons and interests in the research topic	2.5
**Domain 2: study design**		
Theoretical framework		
9. Methodological orientation and Theory	What methodological orientation was stated to underpin the study? e.g. grounded theory, discourse analysis, ethnography, phenomenology, content analysis	N/A
Participant selection		
10. Sampling	How were participants selected? e.g. purposive, convenience, consecutive, snowball	2.4
11. Method of approach	How were participants approached? e.g. face-to-face, telephone, mail, email	2.4
12. Sample size	How many participants were in the study?	3.4.1
13. Non-participation	How many people refused to participate or dropped out? Reasons?	3.4.1
Setting		
14. Setting of data collection	Where was the data collected? e.g. home, clinic, workplace	2.4
15. Presence of non-participants	Was anyone else present besides the participants and researchers?	2.4

16. Description of sample	What are the important characteristics of the sample? e.g. demographic data, date	3.4.1
Data collection		
17. Interview guide	Were questions, prompts, guides provided by the authors? Was it pilot tested?	2.4 and Appendix C
18. Repeat interviews	Were repeat interviews carried out? If yes, how many?	N/A
19. Audio/visual recording	Did the research use audio or visual recording to collect the data?	2.4
20. Field notes	Were field notes made during and/or after the interview or focus group?	2.4
21. Duration	What was the duration of the interviews or focus group?	2.4
22. Data saturation	Was data saturation discussed?	4.3
23. Transcripts returned	Were transcripts returned to participants for comment and/or correction?	2.4
**Domain 3: analysis and findings**		
Data analysis		
24. Number of data coders	How many data coders coded the data?	2.4
25. Description of the coding tree	Did authors provide a description of the coding tree?	N/A
26. Derivation of themes	Were themes identified in advance or derived from the data?	2.4
27. Software	What software, if applicable, was used to manage the data?	2.4
28. Participant checking	Did participants provide feedback on the findings?	N/A
Reporting		
29. Quotations presented	Were participant quotations presented to illustrate the themes/findings? Was each quotation identified? e.g. participant number	3.4.2 to 3.4.5
30. Data and findings consistent	Was there consistency between the data presented and the findings?	3.4.2 to 3.4.5
31. Clarity of major themes	Were major themes clearly presented in the findings?	3.4.2 to 3.4.5
32. Clarity of minor themes	Is there a description of diverse cases or discussion of minor themes?	N/A

APPENDIX B – Summary of characteristics extracted

Data on the following specific review characteristics were extracted.

Characteristics of living status:

- Number of updates
- Current living status (living or static)
- Intended updating schedule (e.g. fixed-interval or change to evidence base)

Communication of living methods:

- Location of first mention of LSR approach
- Whether new evidence yet to be included was flagged for the reader (for evidence identified between submission and publication and post-publication)
- Whether the latest version of updated reviews described how conclusions changed from the previous version

Publication cycle speed:

- Search date
- Submission date
- Publication date

APPENDIX C – Interview outline and questions

At the outset of the interview we established the level of experience and expertise of the interviewees by asking them how long they had been involved in evidence synthesis and what their main role was in the living review(s).

The planned interview questions were:

1. What were the main challenges you encountered during the different stages of creating and maintaining your LSR? For each of these challenges, what was the impact, how were they addressed and were they resolved (why/why not)?
    a. Planning
    b. Evidence surveillance
    c. Evidence synthesis
    d. Reporting
    e. Publishing
    f. Updating
2. What parts of the LSR process did you find to be the easiest or most efficient?
3. Conversely, what are the most difficult/complex aspects of conducting an LSR?
4. What factors influenced your decisions about review scope and methods and any change to these between updates?
5. Do you feel your LSR achieved what you wanted it to achieve? Why/why not?
6. Knowing what you know now, what would you do differently next time or what advice would you give others about to embark on a living review? *You may like to consider the roles that people, processes and technologies played in your LSR experience.*
7. Since being involved in an LSR, do you believe they are:
    a. Less or more efficient than traditional systematic reviews? Why?
    b. A realistic future for evidence syntheses?
8. Did you consult guides or handbooks specific to living reviews, for example Cochrane's LSR guidance? If so:
    a. Which ones?
    b. What did you find helpful?
    c. What could be improved?
    d. What was missing?
9. Is there anything else you would like to add or say about your experiences with LSRs?

LSR	Protocol publication location	Location of first mention of LSR approach	Phrase used in first mention of LSR approach	Planned search frequency	Planned screening frequency	Updating triggers	Timing/criteria for exiting living state	Described how conclusions changed	Made new evidence identified pre-publication visible to readers	Made new evidence identified post-publication visible to readers
Nirmatrelvir combined with ritonavir for preventing and treating COVID-19	Cochrane	Abstract	"living systematic review"	Monthly	Immediately	Cochrane criteria*	None provided	No update as of June 2023	Yes	Yes (OSF spreadsheet)
Convalescent plasma for people with COVID-19: a living systematic review	OSF	Title	"living systematic review"	Monthly	Unspecified	"[We will] update the review when we identify relevant new evidence"	None provided	Yes, conclusions changed	Yes	No
Hyperimmune immunoglobulin for people with COVID-19	Cochrane	Abstract	"living systematic review"	Monthly	Immediately	Cochrane criteria	Cochrane criteria**	No update as of June 2023	Yes	No
Ivermectin for preventing and treating COVID-19	Cochrane	Abstract	"living approach"	Monthly	Immediately	Cochrane criteria	None provided	Yes, conclusions changed	Yes	No
Antibiotics for the treatment of COVID-19	PROSPERO	Abstract	"living approach"	Weekly	Immediately	Cochrane criteria	None provided	No update as of June 2023	Yes	No
Systemic corticosteroids for the treatment of COVID-19: Equity-related analysis and update on evidence	PROSPERO	Abstract	"living systematic review"	Weekly	Immediately	Cochrane criteria	None provided	No description how conclusions changed	Yes	No
Inhaled corticosteroids for the treatment of COVID-19	PROSPERO	Abstract	"living systematic review"	Unclear	Immediately	Cochrane criteria	None provided	No update as of June 2023	Yes	No
Colchicine for the treatment of COVID-19	PROSPERO	Abstract	"living systematic review"	Weekly	Immediately	Cochrane criteria	Cochrane criteria	No update as of June 2023	Yes	No
SARS-CoV-2-neutralising monoclonal antibodies for treatment of COVID-19	Cochrane	Abstract	"living systematic review"	Weekly	Immediately	Cochrane criteria	None provided	No update as of June 2023	Yes	No
Interventions for the treatment of persistent post-COVID-19 olfactory dysfunction	Cochrane	Abstract	"living systematic review"	Monthly	Immediately	Fixed-interval schedule (4 monthly)	Cochrane criteria	Yes, conclusions unchanged	Yes	No
Janus kinase inhibitors for the treatment of COVID-19	PROSPERO	Abstract	"living systematic review"	Weekly	Immediately	Cochrane criteria	Cochrane criteria	No update as of June 2023	Yes	No
Remdesivir for the treatment of COVID-19	PROSPERO	Abstract	"living systematic review"	Weekly	Immediately	Cochrane criteria	None provided	Yes, conclusions changed	LSR is static	LSR is static
Vitamin D supplementation for the treatment of COVID-19: a living systematic review	PROSPERO	Title	"living systematic review"	Weekly	Immediately	Cochrane criteria	None provided	No update as of June 2023	Yes	No
Interleukin-6 blocking agents for treating COVID-19: a living systematic review	PROSPERO	Title	"living systematic review"	Daily	Immediately	Fixed-interval schedule (min 6 monthly)	None provided	No description how conclusions changed	LSR is static	LSR is static
Fluvoxamine for the treatment of COVID-19	PROSPERO	Abstract	"living approach"	Weekly	Immediately	Cochrane criteria	None provided	No update as of June 2023	Yes	No
Interleukin-1 blocking agents for treating COVID-19	PROSPERO	Objectives	"living review"	Daily	Immediately	Fixed-interval schedule (min 6 monthly)	None provided	No update as of June 2023	No	No
Efficacy and safety of COVID-19 vaccines	PROSPERO	Plain language summary	"living systematic review"	Weekly	Immediately	Fixed-interval schedule (min 6 monthly)	None provided	No update as of June 2023	No	No
Interventions for the prevention of persistent post-COVID-19 olfactory dysfunction	Cochrane	Background	"living systematic review"	Monthly	Immediately	Fixed-interval schedule (4 monthly)	Cochrane criteria	Yes, conclusions unchanged	Yes	No
SARS-CoV-2-neutralising monoclonal antibodies to prevent COVID-19	Cochrane	Background	"living systematic review"	Weekly	Immediately	Cochrane criteria	None provided	No update as of June 2023	Yes	No

Thoracic imaging tests for the diagnosis of COVID-19	Cochrane	Background	"living systematic review"	Weekly but only described in protocol	Unspecified	None provided	None provided	No description how conclusions changed	LSR is static	LSR is static
Signs and symptoms to determine if a patient presenting in primary care or hospital outpatient settings has COVID-19	Not publicly available	Background	"living systematic review"	Monthly	Unspecified	None provided	None provided	Yes, conclusions changed	LSR is static	LSR is static
Routine laboratory testing to determine if a patient has COVID-19	Cochrane	Background	"living systematic review"	Weekly	Unspecified	Unclear	None provided	No update as of June 2023	No	No
Rapid, point-of-care antigen tests for diagnosis of SARS-CoV-2 infection	Cochrane	Background	"living systematic review"	Weekly but only described in protocol	Unspecified	None provided	None provided	Yes, conclusions changed	Yes	No
Antibody tests for identification of current and past infection with SARS-CoV-2	Cochrane	Background	"living systematic review"	Weekly but only described in protocol	Unspecified	None provided	None provided	Yes, conclusions changed	LSR is static	LSR is static
Risk of thromboembolism in patients with COVID-19 who are using hormonal contraception	Cochrane	Abstract	"living systematic review"	Monthly	Immediately	Fixed-interval schedule (min 6 monthly)	Fixed deadline	No, conclusions unchanged	No	No

*Cochrane's suggested phrasing for updating triggers in non-fixed-interval reviews is "We wait until the accumulating evidence changes our conclusions of the implications of research and practice before republishing the review."

**Cochrane's suggested phrasing for when to exit living mode is "We will regularly review the scope of the review to decide whether to continue or stop updating the review. Decisions to stop may be based on the premise that the conclusions for our main outcomes and populations of interest are unlikely to change with future studies included in the review, no new evidence is expected, or the review question is no longer a priority for policy and practice."

LSR	Domain	Version	Search date	Submission date	Publication date	Days from search to submission	Days from submission to publication	Days from search to publication	Days since previous search	Days since previous publication	Days since most recent publication	Living status	No. of studies included	Study types included	Eligible study designs
Convalescent plasma or hyperimmune immunoglobulin for people with COVID-19: a rapid review		1	23/04/2020	28/04/2020	14/05/2020	5	16	21	This is the first version of the LSR	This is the first version of the LSR			8	7 Case-series; 1 Prospectively planned, single-arm intervention study	Case-series; Cohort studies; Prospectively-planned studies; RCTs
		2	31/05/2020	16/06/2020	10/07/2020	16	24	40	38	57			20	1 RCT; 3 Controlled NRSIs; 16 Non-controlled NRSIs	All
		3	30/08/2020	02/09/2020	12/10/2020	3	40	43	91	94			19	2 RCTs; 8 Controlled NRSIs; 9 Non-controlled NRSIs	All
Convalescent plasma or hyperimmune immunoglobulin for people with COVID-19: a living systematic review		4	17/03/2021	24/03/2021	20/05/2021	7	57	64	199	220			13	12 RCTs; 1 NRSI	RCTs; Non-controlled NRSIs
Convalescent plasma for people with COVID-19: a living systematic review	Treatment	5	03/03/2022	22/06/2022	01/02/2023	111	224	335	351	622	120	Living	33	33 RCTs	RCTs
		1	05/05/2020	06/08/2020	30/09/2020	93	55	148	This is the first version of the LSR	This is the first version of the LSR			84	Unspecified	All
		2	22/06/2020	24/10/2020	26/11/2020	124	33	157	48	57			34	30 Cross-sectional studies; 4 Case-control studies	All
		3	30/09/2020	09/02/2021	16/03/2021	132	35	167	100	110			51	Unspecified	All except for case-control studies
Thoracic imaging tests for the diagnosis of COVID-19	Diagnosis	4	17/02/2021	21/10/2021	16/05/2022	246	207	453	140	426	381	Static	98	Unspecified	All except for case-control studies
		1	27/04/2020	14/05/2020	07/07/2020	17	54	71	This is the first version of the LSR	This is the first version of the LSR			16	12 Cross-sectional studies; 3 Case-control studies; 1 Internet survey	All
		2	15/07/2020	08/12/2020	23/02/2021	146	77	223	79	231			44	Unspecified	All
Signs and symptoms to determine if a patient presenting in primary care or hospital outpatient settings has COVID-19	Diagnosis	3	10/06/2021	18/11/2021	20/05/2022	161	183	344	330	451	377	Static	42	42 Prospective cross-sectional studies	42 Prospective cross-sectional studies
		1	25/05/2020	21/07/2020	26/08/2020	57	36	93	This is the first version of the LSR	This is the first version of the LSR			18	Unspecified	All
		2	16/11/2020	19/2/2021	24/03/2021	95	33	128	175	210			78	Unspecified	All
Rapid, point-of-care antigen tests for diagnosis of SARS-CoV-2 infection	Diagnosis	3	08/03/2021	21/01/2022	22/07/2022	319	182	501	112	485	314	Living	155	Unspecified	All
		1	27/04/2020	29/05/2020	25/06/2020	32	27	59	This is the first version of the LSR	This is the first version of the LSR			54	Unspecified	All
Antibody tests for identification of current and past infection with SARS-CoV-2	Diagnosis	2	30/09/2020	20/04/2022	17/11/2022	567	211	778	156	875	196	Static	178	Unspecified	All
		1	09/03/2022	N/A	09/01/2023	N/A	N/A	306	This is the first version of the LSR	This is the first version of the LSR			5	3 NRSIs; 2 Case-series studies	All
Risk of thromboembolism in patients with COVID-19 who are using hormonal contraception	Aetiology	2	13/03/2023	N/A	15/05/2023	N/A	N/A	63	369	126	17	Living	5	3 NRSIs; 2 Case-series studies	All
		1	16/12/2020	N/A	22/07/2021	N/A	N/A	218	This is the first version [illegible]	This is the first version [illegible]			1	1 RCT	RCTs
Interventions for the prevention of persistent post-COVID-19 olfactory dysfunction	Prevention	2	20/10/2021	N/A	05/09/2022	N/A	N/A	320	308	410	269	Living	5	5 RCTs	RCTs
		1	26/05/2021	07/06/2021	28/07/2021	12	51	63	This is the first version of the LSR	This is the first version of the LSR			14	14 RCTs	RCTs
Ivermectin for preventing and treating COVID-19	Treatment & Prevention	2	16/12/2021	04/02/2022	21/06/2022	50	137	187	204	328	345	Living	11	11 RCTs	RCTs
Systemic corticosteroids for the treatment of COVID-19: Equity-		1	16/04/2021	08/06/2021	16/08/2021	53	69	122	This is the first version of the LSR	This is the first version of the LSR			11	11 RCTs	RCTs

LSR	Domain	Version	Search date	Submission date	Publication date	Days from search to submission	Days from submission to publication	Days from search to publication	Days since previous search	Days since previous publication	Days since most recent publication	Living status	No. of studies included	Study types included	Eligible study designs
treatment of COVID-19: Equity-related analysis and update on evidence	Treatment	2	06/01/2022	29/04/2022	17/11/2022	113	202	315	265	458	196	Living	16	16 RCTs	RCTs
		1	16/12/2020	N/A	22/07/2021	N/A	N/A	218	This is the first version of the LSR	This is the first version of the LSR			1	1 RCT	RCTs
Interventions for the treatment of persistent post-COVID-19 olfactory dysfunction	Treatment	2	20/10/2021	N/A	05/09/2022	N/A	N/A	320	308	410	269	Living	2	2 RCTs	RCTs
		1	16/04/2021	08/06/2021	05/08/2021	53	58	111	This is the first version of the LSR	This is the first version of the LSR			5	5 RCTs	RCTs
Remdesivir for the treatment of COVID-19	Treatment	2	31/05/2022	01/07/2022	25/01/2023	31	208	239	410	538	127	Static	9	9 RCTs	RCTs
		1	26/02/2021	14/12/2020	18/03/2021	-74	94	20	This is the first version of the LSR	This is the first version of the LSR			10	10 RCTs	RCTs
Interleukin-6 blocking agents for treating COVID-19: a living systematic review	Treatment	2	07/06/2022	29/09/2022	01/06/2023	114	245	359	466	805	0	Static	32	32 RCTs	RCTs
Antibiotics for the treatment of COVID-19	Treatment	1	14/06/2021	29/07/2021	22/10/2021	45	85	130	This is the first version of the LSR	This is the first version of the LSR	587	Living	11	11 RCTs	RCTs
Hyperimmune immunoglobulin for people with COVID-19	Treatment	1	31/03/2022	16/05/2022	26/01/2023	46	255	301	This is the first version of the LSR	This is the first version of the LSR	126	Living	5	5 RCTs	RCTs
Nirmatrelvir combined with ritonavir for preventing and treating COVID-19	Treatment	1	11/07/2022	03/06/2022	20/09/2022	-38	109	71	This is the first version of the LSR	This is the first version of the LSR	254	Living	1	1 RCT	RCTs
Inhaled corticosteroids for the treatment of COVID-19	Treatment	1	07/10/2021	31/10/2021	09/03/2022	24	129	153	This is the first version of the LSR	This is the first version of the LSR	449	Living	3	3 RCTs	RCTs
Colchicine for the treatment of COVID-19	Treatment	1	21/05/2021	15/07/2021	18/10/2021	55	95	150	This is the first version of the LSR	This is the first version of the LSR	591	Living	4	4 RCTs	RCTs
SARS-CoV-2-neutralising monoclonal antibodies for treatment of COVID-19	Treatment	1	17/06/2021	31/03/2021	02/09/2021	-78	155	77	This is the first version of the LSR	This is the first version of the LSR	637	Living	6	6 RCTs	RCTs
Janus kinase inhibitors for the treatment of COVID-19	Treatment	1	24/02/2022	26/11/2021	13/06/2022	-90	199	109	This is the first version of the LSR	This is the first version of the LSR	353	Living	6	6 RCTs	RCTs
Vitamin D supplementation for the treatment of COVID-19: a living systematic review	Treatment	1	11/03/2021	23/03/2021	24/05/2021	12	62	74	This is the first version of the LSR	This is the first version of the LSR	738	Living	3	3 RCTs	RCTs
Fluvoxamine for the treatment of COVID-19	Treatment	1	01/02/2022	01/03/2022	14/09/2022	28	197	225	This is the first version of the LSR	This is the first version of the LSR	260	Living	2	2 RCTs	RCTs
Interleukin-1 blocking agents for treating COVID-19	Treatment	1	03/11/2021	20/09/2021	26/01/2022	-44	128	84	This is the first version of the LSR	This is the first version of the LSR	491	Living	6	6 RCTs	RCTs
Efficacy and safety of COVID-19 vaccines	Prevention	1	05/11/2021	27/02/2022	07/12/2022	114	283	397	This is the first version of the LSR	This is the first version of the LSR	176	Living	41	41 RCTs	RCTs
SARS-CoV-2-neutralising monoclonal antibodies to prevent COVID-19	Prevention	1	27/04/2022	13/12/2021	17/06/2022	-135	186	51	This is the first version of the LSR	This is the first version of the LSR	349	Living	4	4 RCTs	RCTs

LSR	Domain	Version	Search date	Submission date	Publication date	Days from search to submission	Days from submission to publication	Days from search to publication	Days since previous search	Days since previous publication	Days since most recent publication	Living status	No. of studies included	Study types included	Eligible study designs
Routine laboratory testing to determine if a patient has COVID-19	Diagnosis	1	04/05/2020	03/09/2020	19/11/2020	122	77	199	This is the first version of the LSR	This is the first version of the LSR	924	Living	21	Unspecified	Cross-sectional studies; Case-control studies; Consecutive series

N/A is written where Cochrane was unable to provide the review's submission date

Some reviews re-did their search after the submission, causing some days from search to submission calculations to be negative. These reviews were excluded from any analysis of delays between search, submission or publication dates.

Appendix E: characteristics of randomised trials eligible for inclusion

LSR	Search date	Publication date	Number of RCTs already included	Number of extra RCTs				Total number of extra RCTs	Percentage of RCTs acknowledged	Number of extra participants already included	Number of extra participants				Total number of extra participants	Percentage of participants acknowledged
				Awaiting assessment	Before search	Between search and publication	After publication				Awaiting assessment	Before search	Between search and publication	After publication		
Nirmatrelvir combined with ritonavir for preventing and treating COVID-19	11/07/2022	20/09/2022	1	0	0	0	1	1	50%	2246	0	0	0	264	264	89%
Convalescent plasma for people with COVID-19: a living systematic review	03/03/2022	01/02/2023	33	0	0	16	1	17	66%	24861	0	0	3782	25	3807	87%
Hyperimmune immunoglobulin for people with COVID-19	31/03/2022	26/01/2023	5	0	0	1	1	2	71%	947	0	0	18	461	479	66%
Ivermectin for preventing and treating COVID-19	01/04/2022	21/06/2022	11	1	0	7	5	13	46%	3409	114	0	3446	2818	6378	35%
Antibiotics for the treatment of COVID-19	14/06/2021	22/10/2021	11	0	0	2	2	4	73%	11281	0	0	2055	570	2625	81%
Systemic corticosteroids for the treatment of COVID-19: Equity-related analysis and update on evidence	06/01/2022	17/11/2022	16	0	0	2	1	3	84%	9549	0	0	77	754	831	92%
Inhaled corticosteroids for the treatment of COVID-19	07/10/2021	09/03/2022	3	2	0	2	4	8	27%	3607	96	0	323	1682	2101	63%
Colchicine for the treatment of COVID-19	21/05/2021	18/10/2021	4	0	0	2	13	15	21%	16013	0	0	379	6545	6924	70%
SARS-CoV-2-neutralising monoclonal antibodies for treatment of COVID-19	17/06/2021	02/09/2021	6	0	0	2	5	7	46%	17495	0	0	42	2982	3024	85%
Janus kinase inhibitors for the treatment of COVID-19	24/02/2022	13/06/2022	6	1	0	1	6	8	43%	11145	100	0	432	1033	1565	88%
Vitamin D supplementation for the treatment of COVID-19: a living systematic review	11/03/2021	24/05/2021	3	0	0	0	14	14	18%	356	0	0	0	1915	1915	16%
Fluvoxamine for the treatment of COVID-19	01/02/2022	14/09/2022	2	0	0	2	1	3	40%	1649	0	0	713	1288	2001	45%
Interventions for the treatment of persistent post-COVID-19 olfactory dysfunction	20/10/2021	05/09/2022	2	0	1	10	11	22	8%	30	0	64	621	1428	2113	1%
Interleukin-1 blocking agents for treating COVID-19	05/11/2021	26/01/2022	6	0	0	1	4	5	55%	2132	0	0	30	446	476	82%
SARS-CoV-2-neutralising monoclonal antibodies to prevent COVID-19	27/04/2022	17/06/2022	4	0	0	1	0	1	80%	9749	0	0	2317	0	2317	81%
Interventions for the prevention of persistent post-COVID-19 olfactory dysfunction	20/10/2021	05/09/2022	5	0	0	0	0	0	100%	691	0	0	0	0	0	100%
**Totals**	**-**	**-**	**118**	**4**	**1**	**49**	**69**	**123**	**49%**	**115160**	**310**	**64**	**14235**	**22211**	**36820**	**76%**
